# Structure-Dependent Triglyceride Protection of Freeze-Dried *Lactiplantibacillus plantarum*: Storage Protection Window, Lipid Oxidation, and Stress Adaptation

**DOI:** 10.3390/foods15172952

**Published:** 2026-08-22

**Authors:** Zheneng Sun, Nan Zhang, Xiaomai Wang, Xinyao Wei, Samet Ozturk, Shuxiang Liu

**Affiliations:** 1 College of Food Science, Sichuan Agricultural University, Ya’an 625014, China; 2 College of Biological Science and Engineering, Fuzhou University, Fuzhou 350108, China; 3 Department of Food Engineering, Gümüşhane University, Gümüşhane 29100, Türkiye

**Keywords:** triglycerides, *Lactiplantibacillus plantarum*, probiotic stability, lipid oxidation, stress tolerance, transcriptomics

## Abstract

Lipid-rich matrices can protect probiotics by limiting direct exposure to moisture, oxygen, and gastrointestinal stressors. However, triglycerides are chemically dynamic during storage, and differences in fatty acid structure and oxidation susceptibility may determine whether they exert protective or detrimental effects. In this study, tricaprylin, triolein, and trilinolein were evaluated as post-drying lipid storage matrices for freeze-dried *Lactiplantibacillus plantarum* 124 during storage at 25 °C, and 37 °C was used to accelerate lipid oxidation. Survival during storage, fatty acid chain retention, oxidation-derived products, stress tolerance, qualitative confocal imaging of membrane-integrity patterns, and transcriptomic responses were analyzed. After 30 d at 25 °C, log reductions were 0.30, 0.24, and 0.24 log10 units in the tricaprylin, triolein, and trilinolein groups, respectively, compared with 0.60 log10 units in the oil-free control, defining a common protective window. Tricaprylin showed the highest fatty acid chain retention, whereas trilinolein exhibited the greatest loss and strongest oxidative change. GC–MS detected no representative oxidation-derived products in tricaprylin, while unsaturated triglycerides generated dimethyl azelate and methyl 9-oxononanoate. Cells recovered from the protective window showed improved acid and bile tolerance, with survival increasing from 30.48% to 53.43–55.65% and from 14.85% to 22.49–27.79%, respectively. Confocal imaging further showed better-preserved membrane-integrity patterns in the triglyceride-treated groups after acid exposure. Transcriptomics indicated enhanced transport, redox, DNA-related, and cell-envelope responses, accompanied by reduced translation and growth-associated metabolism. Within the experimental conditions tested, these results suggest that triglyceride-associated attenuation of viability loss varies among triglyceride systems and across storage stages, while excessive oxidation of unsaturated triglycerides may weaken probiotic stability.

## 1. Introduction

Probiotic functionality depends on the survival of sufficient numbers of viable, physiologically active cells during processing, storage, and gastrointestinal exposure [1]. During these stages, probiotic bacteria encounter multiple stresses, including dehydration, oxygen exposure, acid, bile salts, and storage-associated environmental changes. During gastrointestinal transit, probiotic survival is supported by coordinated stress responses involving intracellular pH homeostasis, membrane and cell-envelope adaptation, molecular chaperones and repair systems, detoxification and efflux mechanisms, and metabolic regulation [2,3,4]. Food matrices can strongly influence probiotic stability by shaping the local physicochemical environment surrounding bacterial cells. Lipid-rich foods, such as chocolate and cheese, have been reported to support probiotic viability during storage and simulated gastrointestinal digestion [5,6]. Cheese-based systems are often favorable because of their buffering capacity, relatively low oxygen conditions, and structured matrix, while chocolate and other fat-containing complex foods may provide low water availability and physical protection during digestion [7,8]. These findings suggest that lipids may contribute not only as nutritional components but also as functional matrix elements involved in maintaining microbial viability.

Among lipid components, triacylglycerols (triglycerides) are particularly relevant because they are the major neutral lipid fraction of edible oils and can form structurally different lipid environments. Previous studies have documented the survival of lactobacilli in different lipid-containing matrices, including *Lacticaseibacillus paracasei* ATCC 55544 in whole-milk powder, *Lacticaseibacillus rhamnosus* GG in full-fat and reduced-fat peanut butter, and *Lactobacillus acidophilus* in cocoa-butter microparticles [9,10,11]. These findings support the potential role of lipid matrices in maintaining bacterial viability, although the effects depend on matrix composition and storage temperature. Medium-chain or structured triglycerides have been incorporated into probiotic chocolate spreads, where *Enterococcus faecium* CRL 183 maintained stable viability during 180 days of storage, and the product fatty acid profile was improved [12]. Lipid-based encapsulation systems, including solid lipid microparticles, have also been shown to improve probiotic viability and stability under stressed conditions [13]. The protective contribution of triglycerides may be associated with the formation of hydrophobic and low-moisture microenvironments around bacterial cells, which can reduce direct exposure to water, oxygen, and other mobile stressors. In addition, bacterial membrane properties are closely related to stress tolerance: *Lacticaseibacillus casei* maintains membrane functionality under acid stress, and changes in fatty acid composition and membrane fluidity have been associated with improved tolerance in lactic acid bacteria [14,15]. Accordingly, we used structurally defined triglycerides to investigate how lipid chain length, unsaturation, and oxidation state influence the storage protection and subsequent physiological responses of freeze-dried cells.

However, lipid-containing systems are not chemically static during storage. Unsaturated triglycerides, such as triolein and trilinolein, can undergo autoxidation and generate hydroperoxide triglycerides as well as oxidized derivatives during storage or heating [16]. More recent lipidomic studies further show that triglyceride structure affects the formation of non-volatile oxidation products, including epoxy-, hydroxy-, and oxo-triglyceride derivatives [17,18]. Heating of triolein and trilinolein can also induce epoxy fatty acid formation and cis–trans isomerization. Linoleic acid triglycerides may undergo isomerization and degradation reactions that generate conjugated or non-conjugated isomers and cleavage products [19,20,21]. These storage- or oxidation-related changes may alter the local environment around bacterial cells and influence their tolerance. Importantly, this influence should not be interpreted solely as deterioration. Studies on lactic acid bacteria indicate that sublethal stress can induce adaptive or cross-protective responses, often accompanied by changes in membrane fatty acid composition, antioxidant capacity, and membrane integrity [15,22,23]. Thus, oxidized lipid systems may have stage-dependent effects, reflecting a balance among matrix protection, lipid structure, and oxidation-related cellular adaptation or injury.

Lipid-rich matrices, lipid oxidation, and probiotic stress tolerance have each been investigated separately. However, the relationships among triglyceride structure, degree of oxidation, and probiotic acid and bile tolerance remain insufficiently characterized within a unified experimental system. *L. plantarum* 124 was originally isolated from the gut microbiota of centenarians. This strain has previously shown antioxidant potential and, in animal studies, anti-inflammatory, oxidative-stress-relieving, and intestinal-barrier-protective effects, partly attributed to its metabolite mesaconic acid [24]. In this study, *L. plantarum* 124 was used as a model probiotic strain. Three structurally defined triglycerides, tricaprylin, triolein, and trilinolein, were selected to represent differences in chain length, unsaturation degree, and oxidation susceptibility. This design is consistent with the need to compare physical lipid protection and oxidation-associated responses under the same stress conditions. The objectives of this study were: (i) to characterize the fatty acid chain retention and oxidation-related changes in these triglycerides at selected storage stages; (ii) to compare the effects of triglyceride structure and storage condition on the acid, bile salt, and simulated gastrointestinal fluid tolerance of *L. plantarum* 124; and (iii) to identify the effective protective window of the lipid systems and to explore whether the observed effects were associated with microenvironmental protection, lipid structure, or oxidation-related adaptive responses.

## 2. Materials and Methods

### 2.1. Materials, Triglycerides, Bacterial Strain, and Culture Media

Tricaprylin (≥97%, CAS 538-23-8) was purchased from Shanghai Yuanye Bio-Technology Co., Ltd. (Shanghai, China). Triolein (≥97%, CAS 122-32-7) was purchased from Sigma-Aldrich (Shanghai) Trading Co., Ltd. (Shanghai, China). Trilinolein (95%, CAS 537-40-6) was purchased from Shanghai Macklin Biochemical Technology Co., Ltd. (Shanghai, China). A C17:0 fatty acid methyl ester (FAME) internal standard stock solution was prepared at 1 mg/mL in methanol from Solarbio Science & Technology Co., Ltd. (Beijing, China). De Man, Rogosa and Sharpe broth (MRS broth) and MRS agar were purchased from Qingdao Hope Bio-Technology Co., Ltd. (Qingdao, China). Phosphate-buffered saline (PBS, pH 7.4) was purchased from Shanghai Acmec Biochemical Technology Co., Ltd. (Shanghai, China). Tween 80, hydrochloric acid, BF_3_-methanol, n-hexane, and methanol (all chromatographic grade) were obtained from Beijing Solarbio Science & Technology Co., Ltd. (Beijing, China). Bovine bile salts, pepsin, and trypsin were also obtained from the same supplier.

*L. plantarum* 124 was provided by the Institute of Microbiology, Guangdong Academy of Sciences. The strain was stored at −80 °C in MRS broth containing 25% (*v*/*v*) glycerol.

### 2.2. Preparation of Freeze-Dried L. plantarum 124 Powder and Determination of Initial Viable Counts

Frozen glycerol stocks of *L. plantarum* 124 were thawed at room temperature for 3–5 min. The thawed culture was streaked onto MRS agar plates and incubated at 37 ± 2 °C for 48 h for colony isolation [25]. From isolated colonies, three independent culture preparations were established. Each preparation was grown separately in sterile MRS broth to obtain sufficient biomass for gram-scale freeze-dried bacterial powder. All three cultures were cultivated concurrently under identical conditions and were processed independently throughout all subsequent steps. After cultivation, each culture preparation was distributed into 50 mL centrifuge tubes (≤35 mL per tube) and centrifuged at 6000 rpm and 4 °C for 10 min using an X3R centrifuge (Thermo Fisher Scientific Inc., Shanghai, China). The supernatant was discarded. Within each biological replicate, cell pellets from all tubes were combined; material from different replicates was never pooled. Each cell pellet was washed three times with sterile 0.1 mol/L phosphate-buffered saline by resuspension and centrifugation to remove residual medium.

After the final wash, the cell pellet was thoroughly mixed with a freeze-drying protective solution containing 4% (*w*/*v*) monosodium glutamate, 10% (*w*/*v*) sucrose, and 5% (*w*/*v*) fructooligosaccharides at a ratio of 1 g wet cell pellet to 2 mL protective solution. Monosodium glutamate was included as a component of the mixed lyoprotectant because previous studies have demonstrated its protective effects during freeze-drying and storage of lactic acid bacteria [26,27,28]. The resulting mixtures were rapidly frozen in liquid nitrogen [29,30]. The three independently prepared samples were placed in a freeze dryer and vacuum freeze-dried simultaneously during the same 48-h cycle (LGJ-18S freeze-dryer, Beijing Songyuan Huaxing Technology Development Co., Ltd., Beijing, China). The resulting three freeze-dried *L. plantarum* 124 powder preparations were separately collected, purged with nitrogen, sealed, and stored under dry conditions until further use [31]. Water activity (a_w_) of the freeze-dried powder was measured at 25 °C (AQUALAB 4TE, METER Group, Inc., Pullman, WA, USA) and confirmed to be below 0.20 after stabilization.

For initial viable counts (N_0_), 0.5 g of freeze-dried powder was suspended in 10 mL sterile PBS and vortexed for 30 s. The suspension was held at room temperature for approximately 20 min to allow full rehydration and recovery of metabolic activity. Initial viable counts (N_0_) were determined using the spread-plate technique (standard plate count method) [32]. The suspension was vortexed, and 1 mL was transferred into 9 mL sterile PBS to prepare a 10^−1^ dilution; serial 10-fold dilutions were then made up to 10^−9^. For each dilution, 100 μL was plated onto MRS agar and incubated at 37 °C for 48 h. Only plates yielding 30 to 300 colonies were counted. Viable counts were first calculated as CFU/mL of the rehydrated suspension according to the dilution factor and plated volume [33]. Conversion to CFU per gram of bacterial powder equivalent is described in Section 2.3.

### 2.3. Storage Experiments, Accelerated Storage, and Protective-Window Selection

All samples were prepared from freeze-dried *L. plantarum* 124 powder produced with the same mixed lyoprotectant formulation (Section 2.2). For each triglyceride-treated group, 5 g of powder was mixed with 5 g of tricaprylin, triolein, or trilinolein (1:1 mass ratio, 10 g total). The oil-free control consisted of 5 g of the same freeze-dried powder without triglyceride addition. Thus, the lyoprotectant background was identical, and the only variable was the presence and type of triglyceride. For each of the three biological replicates described in Section 2.2, the freeze-dried bacterial powder was separately assigned to the oil-free control and the three triglyceride-treated groups. Each treatment replicate was then divided into two portions for storage at 25 °C and 37 °C, yielding eight treatment–temperature combinations, each with *n* = 3.

Separate sealed desiccators were equilibrated at 25 °C and 37 °C using saturated lithium chloride solutions containing excess undissolved salt, following the constant-humidity principle of ASTM E104 [34]. When hygrometer readings stabilized at approximately 11.3% and 11.2% RH, respectively, samples were placed in open Petri dishes inside the desiccators. The desiccators were kept under ambient air without nitrogen purging, and relative humidity was monitored throughout the 90-day storage period in temperature-controlled chambers (Bluepard, Chengdu Meisheng Technology Co., Ltd., Chengdu, China). Samples were collected every 15 days (15, 30, 45, 60, 75, 90 d). The 25 °C condition served as a mild ambient-storage condition representative of room-temperature stability testing of freeze-dried probiotics [35,36], while 37 °C was used as accelerated storage to promote lipid oxidation [37,38].

For viable-count determination, 0.5 g of oil-free powder or 1.0 g of triglyceride-treated sample was suspended in 10 mL of sterile PBS containing 0.05% (*v*/*v*) Tween 80. Because the bacterial powder and triglyceride were mixed at a 1:1 mass ratio, 1.0 g of the mixture contained ~0.5 g of bacterial powder; thus, all samples represented the same bacterial powder equivalent. The suspension was vortexed for 30 s and held at room temperature for 30 min to allow rehydration and recovery of metabolic activity. Viable counts were first determined as CFU/mL of the rehydrated suspension using the spread-plate method described in Section 2.2. For storage analysis, the viable counts were normalized to bacterial powder equivalent according to

(1) $$C_{g}=\frac{C_{s}V}{m_{eq}}$$

where C_g_ is the viable count expressed as CFU/g bacterial powder equivalent, C_s_ is the viable count in the rehydrated suspension expressed as CFU/mL, V is the total suspension volume (10 mL), and m_eq_ is the bacterial powder equivalent represented by the sample (0.5 g). Accordingly, a single conversion factor of 20 (10 mL/0.5 g) was applied to the CFU/mL values to obtain CFU/g bacterial powder equivalent. No additional mass-to-volume conversion was applied after this normalization. The resulting viable counts were expressed as log CFU/g bacterial powder equivalent. Storage-induced log reduction at sampling time t was calculated as [39]

(2) $$\mathrm{Log}\;\mathrm{reduction}=\log_{10}\left(N_{0}\right)-\log_{10}\left(N_{t}\right)$$

where N_0_ and N_t_ are the viable counts expressed as CFU/g bacterial powder equivalent before storage and at sampling time, respectively. Storage-induced log reduction was expressed in log_10_ units. Because the same conversion factor was applied to both N_0_ and N_t_, normalization to bacterial powder equivalent did not alter the numerical log-reduction values.

Under mild storage conditions (25 °C), the difference in storage-induced log reduction (ΔLR) was calculated as the mean log reduction of the oil-free control minus that of each triglyceride-treated group and is expressed in log_10_ units. Positive ΔLR values indicated smaller storage-induced viability losses than the oil-free control, whereas negative values indicated greater losses. At each storage time, all triglyceride-treated groups were compared with the oil-free control using Dunnett’s multiple-comparison test [40]. The protective-window time point for subsequent stress-tolerance and matrix analyses was selected as the point at which all triglyceride-treated groups showed significantly smaller viability losses and the largest mean positive ΔLR. Storage at 37 °C was used to evaluate accelerated lipid oxidation and to define the boundary between triglyceride-associated attenuation of viability loss and storage-induced damage.

### 2.4. Analysis of Fatty Acid Chain Retention and Oxidation-Derived Products in Triglyceride Systems

Fatty acid methyl ester (FAME) analysis was performed on oil samples collected at three stages: the initial stage (0 h, no storage oxidation), the selected protective-window time point under 25 °C storage, and the corresponding time point under 37 °C accelerated storage. The analysis focused on retention or loss of the original fatty acid chains and the formation of newly detectable lipid degradation products.

Briefly, 50 mg of each oil sample was accurately weighed into a dry stoppered glass tube. Then 2.0 mL of 0.5 mol/L NaOH in methanol was added, and the mixture was refluxed at 65 °C for 30 min for saponification [41]. After slight cooling, 2.0 mL of 14% BF_3_-methanol was added, and the mixture was refluxed again at 65 °C for 30 min for methylation. After cooling to room temperature, 1.0 mL of n-hexane was added for extraction [42]. The tube was vigorously shaken and allowed to stand for phase separation. The upper organic phase was collected and filtered through a 0.22-μm organic membrane prior to GC-MS sample preparation [43].

Because the major FAMEs and degradation products differed substantially in abundance, two injection solutions were prepared from the filtered organic phase. Vial A was used for determination of the major methylated products derived from the original fatty acid chains. For vial A, 50 μL of the extract, 10 μL of C17:0 FAME internal standard stock solution (1 mg/mL, in methanol), and 940 μL of n-hexane were mixed to obtain a final volume of 1000 μL. Vial B was used for determination of newly formed lipid degradation products. For vial B, 400 μL of the extract, 10 μL of diluted C17:0 FAME internal standard solution (0.01 mg/mL), and 590 μL of n-hexane were mixed to obtain a final volume of 1000 μL. Vial A and vial B were analyzed separately for semi-quantification of the major FAMEs and low-abundance degradation products, respectively [44].

GC–MS analysis was performed using an Agilent 8890 gas chromatograph coupled to an Agilent 5977C mass selective detector (MSD) (Agilent Technologies, Santa Clara, CA, USA), equipped with a VF-WAXS capillary column (Agilent Technologies Inc., Santa Clara, CA, USA) (30 m × 0.25 mm × 1.00 μm) [45]. The injector temperature was 250 °C; injection volume, 1 μL; split ratio, 50:1. The oven temperature program was as follows: 100 °C for 2 min; ramped to 180 °C at 10 °C/min and held for 2 min; then ramped to 240 °C at 3 °C/min and held for 10 min. The mass spectrometer was operated in EI mode at 70 eV with a scan range of m/z 40–400. Compound identification was based on retention behavior, NIST mass spectral library matching, information from the literature, and the theoretical plausibility of typical triglyceride oxidation or degradation products [46]. Only compounds meeting these criteria were included in the subsequent semi-quantitative analysis, while low-confidence matches and likely impurity peaks were excluded. Semi-quantification was performed using C17:0 FAME as the internal standard. The relative response value of each compound was calculated as [47]

(3) $$C_{i}=\frac{A_{i}}{A_{IS}}\times \frac{m_{IS}\times V_{ext}}{m_{0}\times V_{aliquot}}$$

where C_i_ is the semi-quantitative content of compound i, A_i_ is the peak area of compound i, A_IS_ is the peak area of the C17:0 FAME internal standard, m_IS_ is the mass of the internal standard added to the injection vial, V_ext_ is the total volume of the organic phase obtained after methylation and extraction, V_aliquot_ is the volume of the organic phase used to prepare the injection solution, and m_0_ is the initial mass of the oil sample. Based on the sample preparation parameters, the major FAMEs in vial A were calculated as (A_i_/A_IS_) × 4 and expressed as μg/mg oil, the low-abundance degradation products in vial B were calculated as (A_i_/A_IS_) × 5 and expressed as μg/g oil. Changes in compound abundance during storage were evaluated by comparing the semi-quantitative contents of the same compound across storage stages.

### 2.5. Preparation of Rehydrated and Oil-Removed Cell Suspensions from Protective-Window Samples

Samples from the selected protective-window condition (25 °C, 30 d) were used for the subsequent assays. To standardize the bacterial powder equivalent across all assays, 0.5 g of oil-free powder or 1.0 g of triglyceride-treated sample (containing ~0.5 g of bacterial powder) was suspended in 10 mL sterile PBS containing 0.05% (*v*/*v*) Tween 80 [48]. The mixture was vortexed for 30 s and left at room temperature for 20–30 min to ensure rehydration and dispersion. External triglycerides were removed by washing the suspension twice with PBS containing 0.1% (*v*/*v*) Tween 80, followed by two washes with sterile PBS to remove residual Tween 80. Each wash consisted of vortexing for 1 min and centrifuging at 6000 rpm and 4 °C for 10 min. The final cell pellet was resuspended in 10 mL sterile PBS and used for subsequent stress-tolerance assays, confocal microscopy, and transcriptomic sample preparation. The oil-free control was processed identically to avoid bias from sample-handling differences.

### 2.6. In Vitro Post-Storage Stress-Tolerance Assays Based on Viable Counts

The rehydrated and oil-removed cell suspension prepared as described in Section 2.5 was used for stress-tolerance assays. For each assay, 1 mL of suspension was inoculated into the stress medium or simulated digestive fluid, vortexed for 30 s, and then divided into independent parallel tubes for the 0-h and 3-h sampling points. The 0-h sample was taken immediately after mixing; the 3-h sample was taken after incubation at 37 ± 2 °C for 3 h. Before each sampling, the tube was vortexed for 30 s, and 1 mL was withdrawn for viable count determination. Serial dilution and plating were performed as described in Section 2.2. Viable counts at 0 and 3 h were determined as CFU/mL of the corresponding liquid challenge suspension and are reported as log10 CFU/mL where applicable [49,50]. Stress tolerance was evaluated using survival percentage calculated as [51]

(4) $$Survival\;percentage\left(\%\right)=\frac{N_{3h}}{N_{0h}}\times 100\%$$

where N_0h_ and N_3h_ are the non-log-transformed viable counts (CFU/mL) measured immediately after inoculation and after 3 h of exposure, respectively. Survival percentage was calculated separately for each biological replicate by normalizing its 3 h viable count to the corresponding 0 h viable count. Because both counts were expressed on the same CFU/mL basis, the units cancelled in the ratio, yielding a dimensionless percentage.

#### 2.6.1. Acid Tolerance Assay

Acid tolerance was assessed in MRS broth adjusted to pH 2.5 with 5% HCl. One milliliter of cell suspension was inoculated into 10 mL of acidified MRS broth, and survival percentage was determined after 3 h of acid exposure [52].

#### 2.6.2. Bile Salt Tolerance Assay

Bile salt tolerance was determined in MRS broth supplemented with 0.3% (*w*/*v*) bovine bile salts. One milliliter of cell suspension was inoculated into 10 mL of bile salt-containing MRS broth, and survival percentage was determined after 3 h of exposure [53].

#### 2.6.3. Simulated Gastric Fluid Tolerance Assay

Simulated gastric fluid was prepared by dissolving pepsin at 3 g/L in sterile PBS, adjusting the pH to 3.0 with HCl, and filtering through a 0.22 μm membrane. For the assay, 1 mL of cell suspension was mixed with 5 mL of simulated gastric fluid and 1.5 mL of 0.5% (*w*/*v*) NaCl solution, and survival percentage was determined after 3 h of exposure [54].

#### 2.6.4. Simulated Intestinal Fluid Tolerance Assay

Simulated intestinal fluid was prepared by dissolving trypsin at 1 g/L and bovine bile salts at 0.1% (*w*/*v*) in sterile PBS. The pH was adjusted to 8.0, and the solution was filtered through a 0.22 μm membrane. For the assay, 1 mL of cell suspension was mixed with 5 mL of simulated intestinal fluid and 1.5 mL of 0.5% (*w*/*v*) NaCl solution, and survival percentage was determined after 3 h of exposure [55].

### 2.7. Qualitative Confocal Visualization of Cell Membrane Integrity Under the Selected Acid-Stress Condition

Cell membrane integrity after pH 2.5 acid stress was qualitatively visualized using SYTO 9/propidium iodide (PI) double staining [56,57]. This condition was selected because, in Section 2.6, all three triglyceride-treated groups showed significantly higher culture-based survival than the oil-free control. Rehydrated and oil-removed cell suspensions prepared as described in Section 2.5 were used without further adjustment of cell concentration. The oil-free control and triglyceride-treated groups were examined at 0 and 3 h of stress exposure. SYTO 9 and PI stock solutions (3.34 and 20 mM, respectively) were mixed 1:1 (*v*/*v*). For staining, 3 μL of the dye mixture was added to 1 mL of sample, yielding final concentrations of ~5 μM SYTO 9 and ~30 μM PI, followed by incubation in the dark at room temperature for 10 min. Samples at 0 h were stained immediately before stress exposure, whereas independent parallel samples for the 3 h observation were collected and stained during the final 15 min of treatment so that imaging coincided with the 3 h endpoint [58]. After staining, 100 μL of each sample was transferred to a glass-bottom dish and observed using a ZEISS LSM 910 confocal laser scanning microscope (Carl Zeiss Microscopy GmbH, Jena, Germany) equipped with a 63× oil-immersion objective. SYTO 9 and PI were excited at 488 and 561 nm, respectively, and acquired by sequential scanning to minimize spectral crosstalk [59]. Representative fields were recorded for qualitative comparison of membrane-integrity patterns among groups. For descriptive assessment of the 3 h endpoint, identifiable cells within each representative field were additionally classified as PI-positive or PI-negative according to the presence or absence of detectable PI-associated fluorescence. Cells showing clear PI-associated red fluorescence or overlapping yellow/orange fluorescence were classified as PI-positive. The proportions of PI-positive and PI-negative cells were calculated relative to the total number of identifiable cells within the corresponding field.

### 2.8. Transcriptomic Sequencing of Rehydrated and Oil-Removed Cells from Protective-Window Samples

Transcriptomic sequencing was performed using samples from the selected protective-window condition (25 °C, 30 d), including the oil-free control and all three triglyceride-treated groups, with three independent biological replicates per group. Rehydrated and oil-removed cell suspensions were prepared as described in Section 2.5. The suspensions were centrifuged at 6000 rpm and 4 °C for 10 min, and the cell pellets were snap-frozen in liquid nitrogen and stored at −80 °C until RNA extraction [60]. RNA was extracted after the standardized rehydration and oil-removal procedure, before any subsequent stress challenge. Thus, the RNA-seq profiles represent the post-storage, post-rehydration state of the cells, not the transcriptional state during dry storage or subsequent stress challenges.

Total RNA was extracted using the TRIzol method according to the manufacturer’s instructions [61]. RNA quality and integrity were assessed using an Agilent 2100 Bioanalyzer (Agilent Technologies Inc., Santa Clara, CA, USA) [62]. Ribosomal RNA was removed to enrich bacterial mRNA. The enriched mRNA was then fragmented into short fragments. First-strand cDNA was synthesized using random hexamer primers and reverse transcriptase, followed by second-strand cDNA synthesis using DNA polymerase I and RNase H. The double-stranded cDNA was purified, end-repaired, A-tailed, and ligated to adapters to construct sequencing libraries. Libraries were sequenced on an Illumina NovaSeq platform (Illumina, Inc., San Diego, CA, USA) with 150-bp paired-end reads [63]. Raw sequencing data were processed to remove adapter-containing reads, paired reads in which either read contained more than 10% ambiguous bases (N), and paired reads in which more than 50% of bases in either read had a Phred quality score ≤ 5. Quality metrics, including Q20, Q30, and GC content, were subsequently calculated. The resulting high-quality clean reads were used for subsequent transcriptomic analysis [64].

### 2.9. Statistical and Bioinformatic Analysis

All experiments were performed with three independent biological replicates, and data are expressed as mean ± standard deviation. Except for transcriptomic analysis, statistics and figures were generated using GraphPad Prism 10. Statistical significance was defined as *p* < 0.05 and indicated as * *p* < 0.05, ** *p* < 0.01, *** *p* < 0.001, **** *p* < 0.0001, and ns for no significant difference [65].

Storage and stress-tolerance data were analyzed by one-way ANOVA followed by Dunnett’s multiple-comparison test with the oil-free control as the reference group [66]. For storage experiments, ΔLR was calculated as the mean log reduction of the oil-free control minus that of each triglyceride-treated group; positive values indicated smaller viability losses and negative values greater losses. At each storage time, all triglyceride-treated groups were compared with the oil-free control using Dunnett’s test. The protective window was selected as the time point at which all triglyceride-treated groups showed significantly smaller log reductions and the largest mean positive ΔLR. Because these analyses were performed separately at each sampling condition rather than using a multifactorial model, differences among triglyceride systems and storage stages are interpreted as condition-specific response patterns rather than formally established structure, stage, or interaction effects.

GC–MS data were semi-quantified using C17:0 FAME as the internal standard; data from vials A and B were analyzed separately, and changes in the same compound across storage stages were compared. Confocal images were qualitatively evaluated based on the distribution of SYTO 9- and PI-associated fluorescence at 0 and 3 h. At the 3 h endpoint, approximate PI-positive and PI-negative cell proportions were additionally estimated descriptively from the representative fields. Because these field-level estimates were not derived from an independently replicated quantitative imaging dataset, they were not subjected to inferential statistical analysis.

For transcriptomic analysis, clean reads were mapped to the *Lactiplantibacillus plantarum* reference genome GCF_009913655.1 (NCBI RefSeq; assembly ASM991365v1) using Bowtie2 v2.3.4.3 with default parameters. The corresponding NCBI RefSeq genome annotation associated with this assembly was used for gene-level annotation. FPKM values were used for visualization, whereas raw counts were analyzed using DESeq2 (v1.51.6). Raw *p*-values were adjusted for multiple testing using the Benjamini–Hochberg method implemented in DESeq2 to control the false-discovery rate. Differentially expressed genes were defined by an adjusted *p*-value < 0.05 and |log_2_FC| ≥ 1.5. GO terms with *p* < 0.05 were considered significantly enriched [67].

## 3. Results and Discussion

### 3.1. Initial Viability of Freeze-Dried L. plantarum 124 Powder and Triglyceride-Mixed Samples

The freeze-dried *L. plantarum* 124 powder showed a high initial viable count of 11.62 ± 0.06 log CFU/g bacterial powder equivalent. After mixing with tricaprylin, triolein, and trilinolein at a 1:1 mass ratio, the viable counts at 0 h, expressed on the same bacterial-powder-equivalent basis, were 11.25 ± 0.21, 11.18 ± 0.17, and 11.13 ± 0.29 log CFU/g bacterial powder equivalent, respectively. These values remained within the same order of magnitude as the oil-free powder and showed no significant difference, indicating that the mixing process did not cause an obvious immediate loss of viability. The comparable initial viable counts among the control and triglyceride-treated samples confirmed that the prepared systems were suitable for subsequent storage experiments. Because sufficient viable cells are required for probiotics to exert beneficial effects, the high initial viability also provided a reliable basis for evaluating triglyceride-associated attenuation of viability loss during storage [68].

### 3.2. Storage Survival Dynamics and Identification of the Triglyceride-Mediated Protective Window

#### 3.2.1. Protective-Window Formation Under Mild Storage at 25 °C

**Figure 1:**
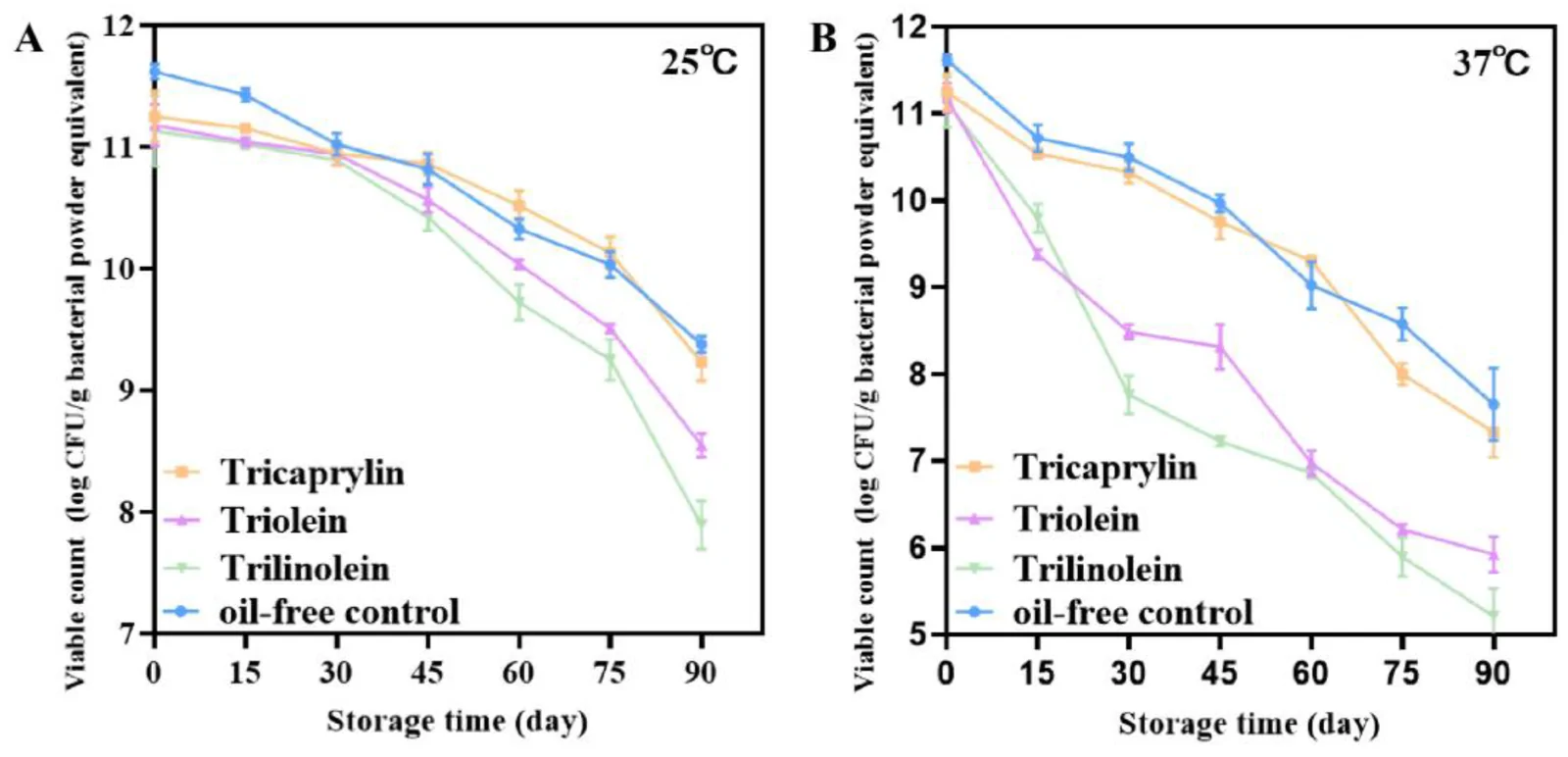
Absolute viable counts of freeze-dried *L. plantarum* 124 in the oil-free control and triglyceride-treated systems during storage. (**A**) Storage at 25 °C. (**B**) Accelerated storage at 37 °C. Viable counts were normalized to the bacterial powder equivalent and expressed as log CFU/g bacterial powder equivalent. Data are presented as mean ± standard deviation from three independent biological replicates (*n* = 3).

Viable counts of freeze-dried *L. plantarum* 124 declined progressively in all groups during storage at 25 °C (Figure 1A). Because the initial viable counts differed slightly among the prepared systems, the storage response was further evaluated using the within-group log reduction relative to the corresponding 0 d value of each group (Table 1). Accordingly, Figure 1 reports the absolute viable-cell level at each time point, whereas the LR values in Table 1 represent the decrease from each group’s own 0 d baseline. The log reduction in the oil-free control increased from 0.19 ± 0.06 log10 units at 15 d to 2.24 ± 0.07 log10 units at 90 d. During the early storage period, the triglyceride-treated groups generally showed smaller within-group viability losses than the oil-free control.

At 15 d, the ΔLR values of tricaprylin, triolein, and trilinolein were positive but relatively small, and triolein did not differ significantly from the oil-free control. At 30 d, the log reductions were 0.30 ± 0.10, 0.24 ± 0.09, and 0.24 ± 0.04 log10 units for the tricaprylin, triolein, and trilinolein groups, respectively, compared with 0.60 ± 0.09 log10 units for the oil-free control. The corresponding ΔLR values were 0.29, 0.36, and 0.36 log10 units, indicating smaller viability losses than in the oil-free control, and all three triglyceride-treated groups differed significantly from the oil-free control. These results identify 25 °C storage for 30 d as a common interval in which the triglyceride-treated groups experienced smaller viability losses relative to their own initial counts, rather than showing a general increase in absolute viable counts. This early-stage reduction in viability loss was consistent with the proposed matrix-level protective role of triglycerides, although the emergence of a common protective interval at 30 d was an experimental observation rather than a predetermined outcome. After 45 d, the responses of the three triglyceride systems gradually diverged. Tricaprylin maintained positive ΔLR values, whereas the effects associated with triolein and trilinolein weakened during prolonged storage and eventually became unfavorable. Based on these results and the protective-window selection criterion defined in the Methods, storage at 25 °C for 30 d was selected as the representative condition for subsequent analyses. This selection was based on the within-group storage-induced log reductions relative to each group’s own day-0 value and the corresponding ΔLR values in Table 1, rather than on between-group differences in the absolute viable counts shown in Figure 1 [69].

#### 3.2.2. Accelerated Viability Loss Under 37 °C Storage

Viable counts declined more rapidly in all groups during storage at 37 °C than at 25 °C (Figure 1B). For the oil-free control, the reduction relative to its own 0 d viable count reached 3.97 ± 0.42 log10 units at 90 d. This result indicates that elevated temperature intensified storage-induced viability loss in the freeze-dried bacterial powder [70].

Under storage at 37 °C, no common protective window was observed among the three triglyceride systems. Tricaprylin showed slightly smaller viability losses than the oil-free control at some time points, but this effect was not consistently maintained. In contrast, triolein and trilinolein showed greater storage-induced losses from the early stage. At 30 d, the log reductions of the triolein and trilinolein groups were 2.69 ± 0.09 and 3.37 ± 0.22 log10 units, respectively, compared with 1.12 ± 0.16 log10 units for the oil-free control. Thus, the unsaturated triglycerides did not improve bacterial survival under accelerated storage and were instead associated with faster inactivation. This difference became more pronounced during prolonged storage, with trilinolein showing the greatest viability loss at 90 d.

Taken together, the effects of triglycerides on bacterial survival depended on storage temperature, duration, and triglyceride structure. A limited interval of smaller within-group viability loss was observed for all three triglyceride-treated groups at 25 °C for 30 d, whereas storage at 37 °C was unfavorable, particularly for the unsaturated triglyceride systems. Therefore, 25 °C storage for 30 d was selected as the representative protective-window condition for subsequent stress-tolerance, lipid oxidation, membrane-integrity, and transcriptomic analyses. This selection represents a condition-specific comparison and should not be interpreted as evidence of a universal protective effect of triglycerides.

### 3.3. Fatty Acid Chain Retention and Oxidation-Derived Products in Triglyceride Systems

The GC–MS total ion chromatograms of the three triglyceride systems at 0 h, 25 °C for 30 d, and 37 °C for 30 d are provided in Supplementary Figure S1. Quantitative analysis of the parent fatty acid methyl esters revealed distinct patterns among the three triglyceride systems, as summarized in Table 2. Tricaprylin showed the highest fatty acid chain retention, with methyl octanoate decreasing only from 942 ± 15 μg/mg oil at 0 h to 912 ± 14 μg/mg oil at 25 °C and 904 ± 4 μg/mg oil at 37 °C, corresponding to retention percentages of 96.8% and 96.0%, respectively. Triolein showed a moderate decrease in methyl oleate, with retention percentages of 94.7% at 25 °C and 85.8% at 37 °C. In contrast, trilinolein exhibited the most pronounced reduction in methyl linoleate, with retention percentages of only 83.3% at 25 °C and 60.8% at 37 °C. These results indicate that fatty acid chain loss increased with the degree of unsaturation, following the order trilinolein > triolein > tricaprylin [71]. Notably, the decrease in parent fatty acid chains was greater than the accumulation of the representative oxidation or cleavage products listed in Table 3, which may be related to the volatilization of some low-molecular-weight oxidation products or their further transformation during storage. This phenomenon also reflects the higher reactivity and temperature sensitivity of unsaturated triglycerides [72,73].

The oxidation- and cleavage-related products detected after storage further supported the different oxidative susceptibilities of the triglyceride systems (Table 3). After excluding low-confidence matches and likely impurity peaks, no representative oxidation-derived products were detected in the tricaprylin system, which was consistent with the high retention of its saturated medium-chain fatty acid. In contrast, triolein generated several fatty acid chain-derived products, including hexanal dimethyl acetal, nonanal dimethyl acetal, methyl nonanoate, and dimethyl azelate, and their abundances were generally higher at 37 °C than at 25 °C [74]. Trilinolein showed stronger oxidation and cleavage signals, including hexanal dimethyl acetal, methyl octanoate, methyl 9-oxononanoate, and dimethyl azelate. Notably, dimethyl azelate increased from 2.86 ± 0.24 μg/g oil at 25 °C to 77.10 ± 6.95 μg/g oil at 37 °C, while methyl octanoate increased from 9.03 ± 0.39 to 61.32 ± 3.67 μg/g oil. These compounds can be regarded as representative signals of unsaturated fatty acid chain oxidation and cleavage, rather than being interpreted simply as direct damaging factors [75]. At the protective-window stage after 30 d of storage at 25 °C, the observed combination of moderate lipid oxidation, smaller viability loss, and subsequent stress-tolerance responses is compatible with a working hypothesis in which the external lipid environment may influence the physiological state of the stored cells through both matrix-related protection and oxidation-associated stress signals. However, the present data do not establish that lipid oxidation products directly induce bacterial adaptation. Under 37 °C accelerated storage, the greater accumulation of oxidation- and cleavage-related products coincided with increased viability loss in the unsaturated triglyceride systems, suggesting that excessive lipid oxidation may be associated with a shift from a potentially protective to an unfavorable storage environment [76].

### 3.4. Stress Tolerance of Rehydrated and Oil-Removed Cells from Protective-Window Samples

**Figure 2:**
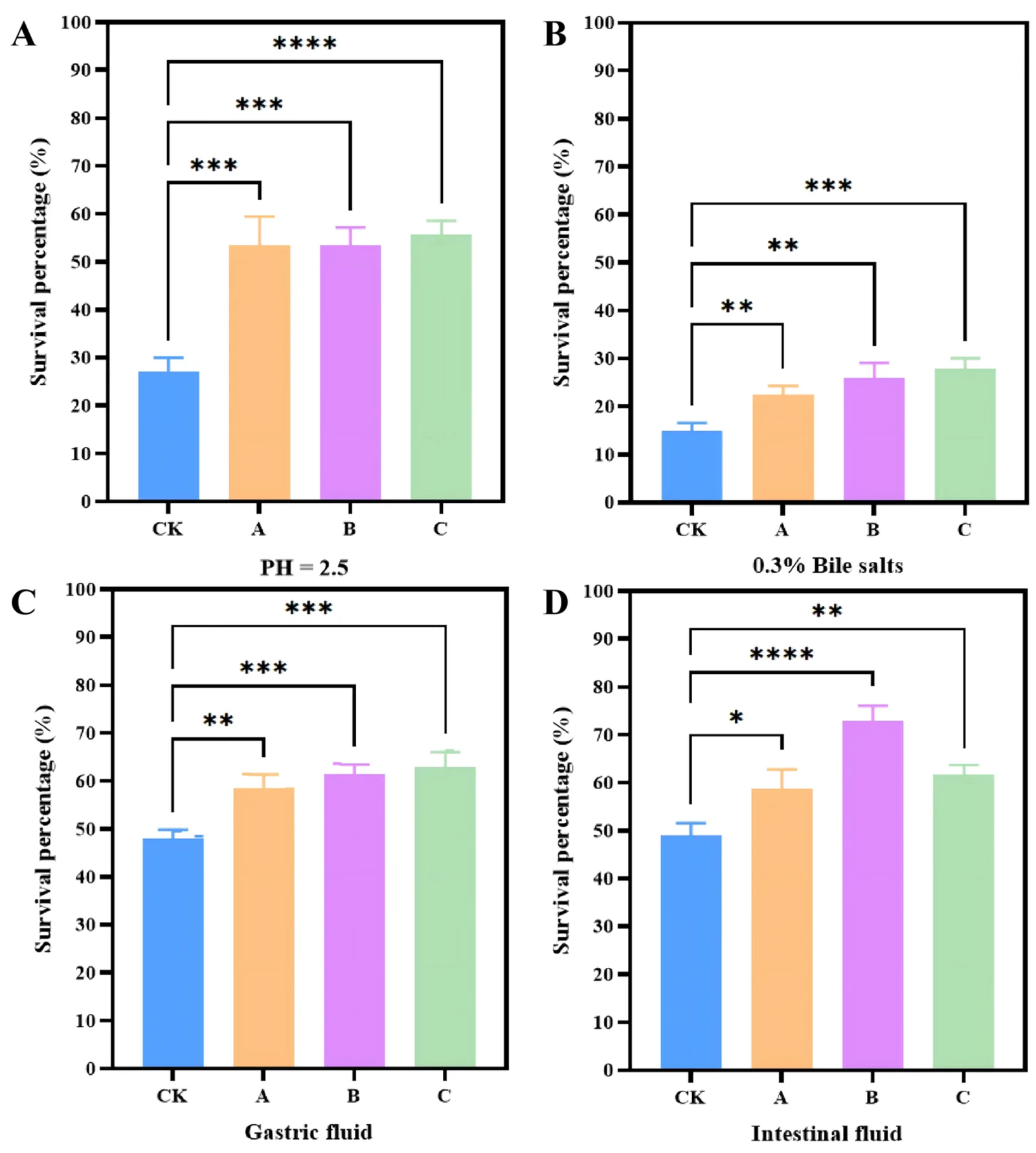
Stress tolerance of rehydrated and oil-removed *L. plantarum* 124 cells obtained from protective-window samples. Survival percentages were determined after 3 h of exposure to (**A**) pH 2.5 acid stress, (**B**) 0.3% bile salts, (**C**) simulated gastric fluid, and (**D**) simulated intestinal fluid. CK denotes the oil-free control. Data are presented as mean ± standard deviation of three independently established and separately processed biological replicates (*n* = 3). Survival percentages were calculated separately for each biological replicate relative to its own corresponding 0 h viable count. Asterisks indicate significant differences between each triglyceride-treated group and CK (* *p* < 0.05, ** *p* < 0.01, *** *p* < 0.001, **** *p* < 0.0001).

The stress tolerance of rehydrated and oil-removed *L. plantarum* 124 cells prepared from the 25 °C 30 d protective-window samples was evaluated under acid, bile salts, simulated gastric fluid, and simulated intestinal fluid conditions (Figure 2). Compared with the oil-free control, all triglyceride-treated groups showed higher survival percentages under the four standardized in vitro challenge conditions, indicating greater post-storage stress tolerance of the recovered cells after removal of the external triglyceride phase. Under pH 2.5 acid stress, the survival percentage of the control was 30.48 ± 3.35%, whereas the survival percentages of the tricaprylin, triolein, and trilinolein groups increased to 53.43 ± 6.01%, 53.50 ± 3.62%, and 55.65 ± 2.90%, respectively, all showing highly significant differences from the control. Under 0.3% bile salt stress, the survival percentage of the control was only 14.85 ± 1.67%, while the triglyceride-treated groups increased to 22.49 ± 1.78%, 25.85 ± 3.21%, and 27.79 ± 2.25%, respectively.

Similar improvement was also observed under simulated digestive conditions. In simulated gastric fluid, the survival percentage increased from 45.99 ± 2.48% in the control to 58.51 ± 2.79%, 61.44 ± 1.97%, and 65.61 ± 3.83% in the tricaprylin, triolein, and trilinolein groups, respectively. In simulated intestinal fluid, the survival percentages of the triglyceride-treated groups were 58.65 ± 4.12%, 72.87 ± 3.19%, and 61.67 ± 2.00%, compared with 48.87 ± 2.68% in the control. Although the magnitude of the differences varied among challenge conditions and triglyceride types, the overall results showed that viable cells recovered from the protective-window samples retained greater tolerance under the standardized in vitro challenge conditions. The coincidence of smaller storage-induced viability losses at 30 d with higher survival in the subsequent stress challenges suggests biological consistency between storage protection and the physiological robustness of the surviving cells. Because the stress-survival percentages were normalized to the corresponding 0 h viable count of each biological replicate, the higher post-storage stress tolerance cannot be explained simply by differences in the number of viable cells entering the challenge assays. The improvement was more consistent under acid stress and simulated gastric fluid, whereas the enhancement under intestinal fluid was more treatment-dependent, with triolein showing the most pronounced effect. Therefore, pH 2.5 acid stress was selected for subsequent qualitative visualization of membrane integrity because all three triglyceride-treated groups showed significantly higher culture-based survival than the oil-free control. The acid, bile salt, and simulated digestive-fluid assays, which are widely used in probiotic formulation, drying, storage, and food-matrix studies, complemented the absolute viable-count data by indicating whether cells recovered from the different triglyceride matrices retained post-storage stress tolerance [50,77]. These results therefore distinguish viable-cell quantity from the physiological robustness of the surviving cells and are interpreted as comparative in vitro stress-tolerance phenotypes rather than direct estimates of survival in vivo.

### 3.5. Qualitative Visualization of Membrane Integrity Under Acid Stress

SYTO 9/PI double staining was used to qualitatively visualize the membrane-integrity patterns of rehydrated and oil-removed *L. plantarum* 124 cells under pH 2.5 acid stress. SYTO 9 stains bacterial nucleic acids and produces green fluorescence, whereas PI preferentially enters cells with increased membrane permeability and produces red fluorescence. Accordingly, predominantly green fluorescence was associated with relatively intact cell membranes, while PI-associated red or yellow/orange fluorescence indicated increased membrane permeability and membrane injury [78,79].

**Figure 3:**
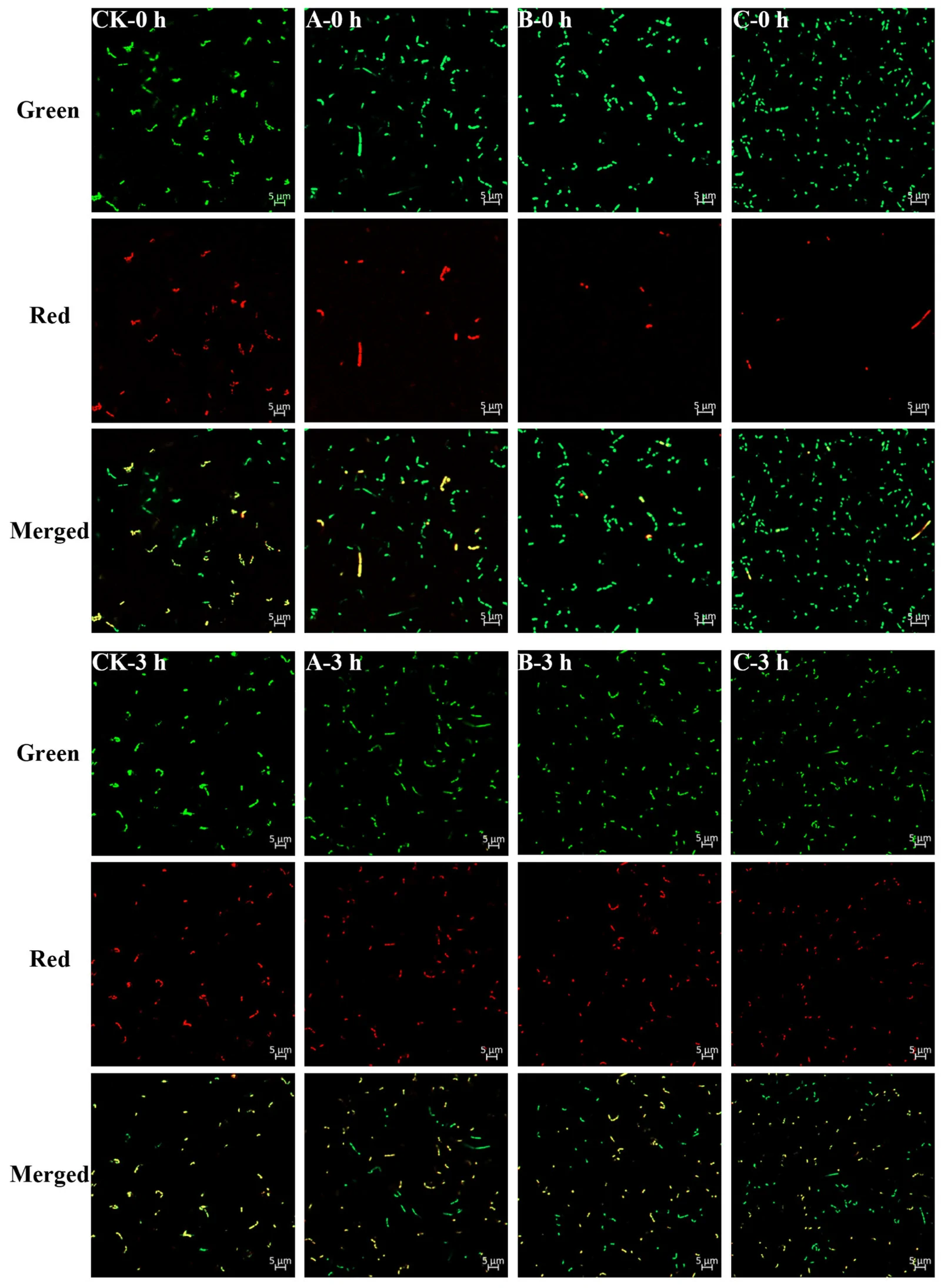
Representative CLSM images showing the membrane-integrity patterns of rehydrated and oil-removed *L. plantarum* 124 cells before and after pH 2.5 acid exposure. Columns represent the oil-free control (CK), tricaprylin (A), triolein (B), and trilinolein (C) groups, with images shown at 0 and 3 h for each group. SYTO 9-associated green fluorescence predominantly represents membrane-intact cells, whereas PI-associated red/yellow fluorescence indicates increased membrane permeability and cellular injury. Images were acquired using a 63× oil-immersion objective. Scale bars = 5 μm.

Because the CLSM samples were prepared using an equivalent bacterial-powder mass and were imaged without further adjustment of cell concentration, the fluorescence images reflected (Figure 3) both the cell populations retained during the preceding 30 d storage and their membrane-integrity status. At 0 h, the oil-free control showed more extensive PI-associated fluorescence than the triglyceride-treated groups, which was consistent with the greater storage-induced viability loss observed in the control before the acid challenge. This baseline pattern was interpreted qualitatively. At the 3 h acid-stress endpoint, descriptive cell-based assessment of the representative fields showed that approximately 82% of identifiable cells in the oil-free control were PI-positive, compared with approximately 56%, 63%, and 62% in the tricaprylin, triolein, and trilinolein fields, respectively. The corresponding approximate PI-negative proportions were 18%, 44%, 37%, and 38%. Thus, at the 3 h endpoint, all three triglyceride-treated representative fields contained a lower proportion of cells with detectable PI-associated membrane permeability than the oil-free control. These field-level percentages are descriptive estimates from the representative images rather than independently replicated quantitative endpoints and were therefore not subjected to statistical comparison. PI positivity is interpreted here as increased membrane permeability rather than as direct evidence of loss of culturability. The image-based observations therefore provide complementary membrane-integrity information to the culture-based acid-survival measurements [80].

### 3.6. Transcriptomic Analysis of Rehydrated and Oil-Removed Cells from Protective-Window Samples

#### 3.6.1. Sequencing Quality and Overall Transcriptomic Variation

Transcriptomic sequencing was performed on rehydrated and oil-removed cells from the oil-free control and the three triglyceride-treated groups after storage at 25 °C for 30 d, corresponding to the representative protective-window condition identified from the storage-survival analysis. The raw RNA-seq data generated in this study have been deposited in the National Center for Biotechnology Information (NCBI) Sequence Read Archive (SRA) under BioProject accession PRJNA1481496. After quality filtering, each sample generated 7.27–7.79 million clean reads and 1.09–1.17 Gb of clean bases. The sequencing error rate was 0.01% for all samples, while the Q20 and Q30 values exceeded 99.55% and 97.73%, respectively. The GC content ranged from 44.71% to 45.95%, and most samples showed total mapping rates above 86%. These results indicated high base-calling accuracy and good mapping quality, supporting subsequent differential expression and functional enrichment analyses [81].

**Figure 4:**
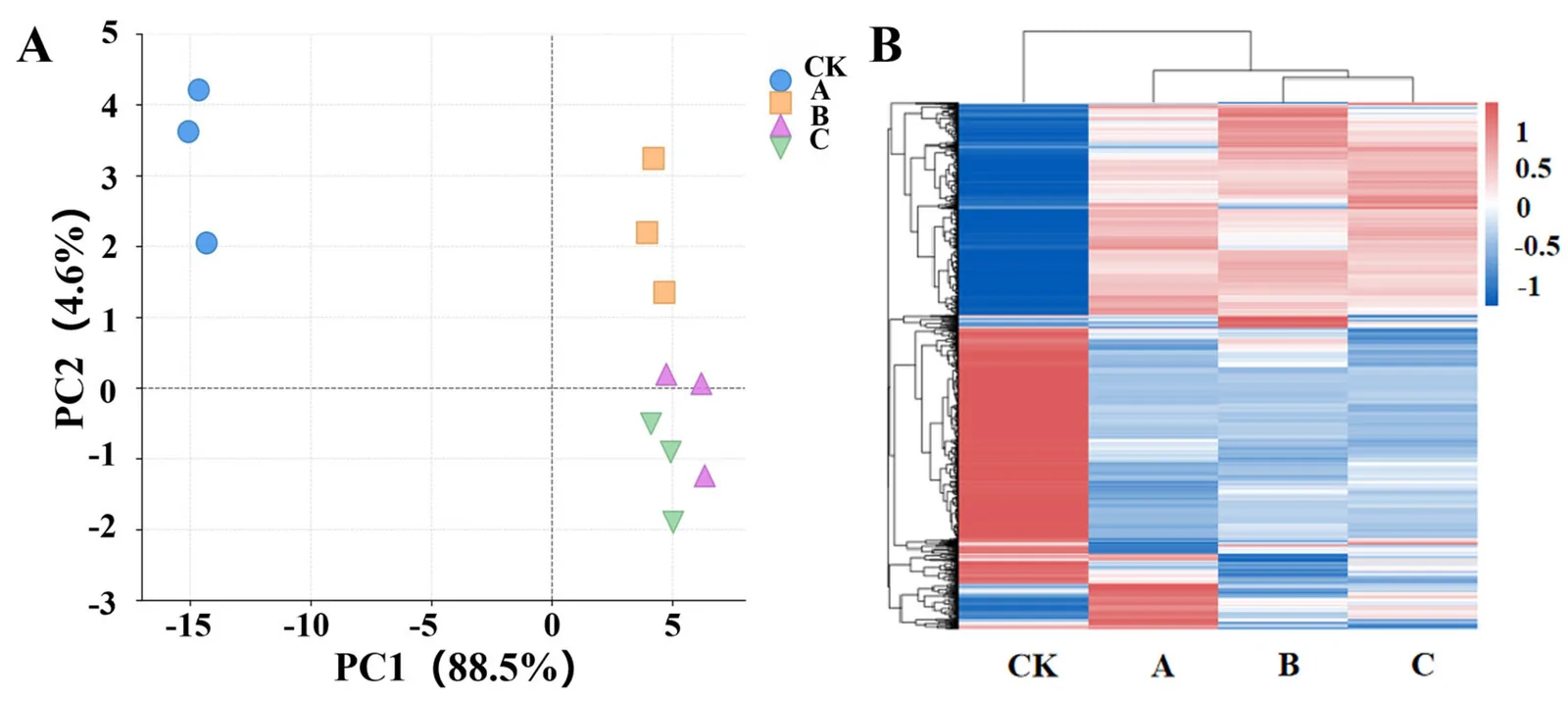
Overall transcriptomic variation in CK and triglyceride-treated groups. (**A**) Principal component analysis (PCA) of transcriptomic profiles. (**B**) Hierarchical clustering heatmap of differentially expressed genes (DEGs). CK, oil-free control; A, tricaprylin; B, triolein; and C, trilinolein.

**Figure 5:**
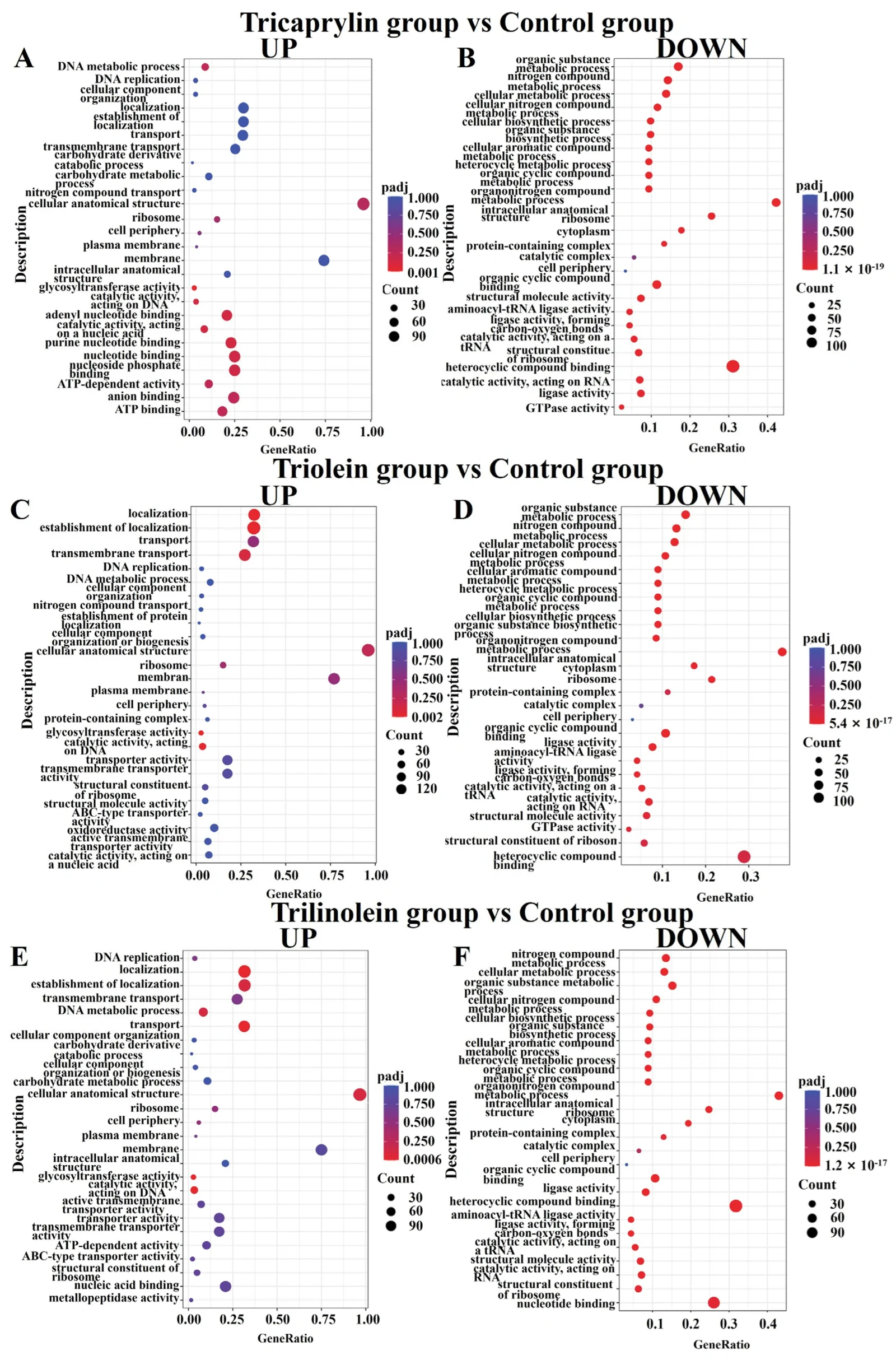
GO enrichment analysis of differentially expressed genes in triglyceride-treated groups compared with the oil-free control. Upregulated and downregulated genes are shown for tricaprylin versus control (**A**,**B**), triolein versus control (**C**,**D**), and trilinolein versus control (**E**,**F**), respectively. Control denotes the oil-free control.

The overall transcriptomic variation among groups was further evaluated by principal component analysis and hierarchical clustering. As shown in Figure 4A, PC1 explained 88.5% of the total variation and clearly separated the oil-free control from the three triglyceride-treated groups, indicating that cells recovered from the different storage matrices displayed distinct transcriptomic profiles after rehydration and oil removal. The three triglyceride-treated groups were distributed on the same side of PC1 but showed partial separation along PC2, suggesting both shared and treatment-specific post-rehydration transcriptional features associated with the different triglyceride-storage conditions. Consistently, the hierarchical clustering heatmap of differentially expressed genes showed that CK formed a distinct expression pattern from the triglyceride-treated groups, whereas A, B, and C displayed partially similar but not identical expression profiles (Figure 4B). Together, these results revealed clear differences in gene-expression patterns associated with the preceding storage conditions, with both shared and triglyceride-specific features among the three triglyceride-treated groups. These differences may reflect distinct cellular responses during recovery and provide a basis for further analysis of GO functional categories associated with upregulated and downregulated genes (Figure 5A–F) [82].

#### 3.6.2. GO Enrichment Analysis and Representative Upregulated Genes in Triglyceride-Treated Groups

GO enrichment analysis of upregulated genes showed that the three triglyceride-treated groups shared enriched terms related to localization, establishment of localization, transport, and transmembrane transport, with these categories more prominent in the triolein and trilinolein groups (Figure 5A,C,E). These terms mainly describe cellular localization and substance transport [83,84]. Consistently, representative upregulated genes included *srlE*, encoding a PTS glucitol/sorbitol transporter subunit IIB, a BCCT family transporter, and a metal ABC transporter ATP-binding protein (Table 4), indicating increased transcription related to carbohydrate, compatible-solute, and ATP-dependent transport [85,86]. At the molecular-function level, transporter activity, active transmembrane transporter activity, and ABC-type transporter activity were also enriched, particularly in the unsaturated triglyceride groups. ABC transporters are ATP-dependent systems involved in nutrient uptake, ion homeostasis, and export of exogenous or unfavorable small molecules [87]. These GO- and gene-level changes were consistent with a transport-related response that may help maintain ion, small-molecule, and metabolite exchange after rehydration and oil removal. This interpretation is also compatible with the higher post-storage survival percentages observed in the triglyceride-treated groups under acid, bile salt, and simulated gastrointestinal challenges, although the transcriptomic samples were collected before these assays. In addition, the stronger fatty acid chain oxidation or cleavage observed by GC–MS in triolein and trilinolein may partly explain the more prominent transport-related response in these groups. The SDR family oxidoreductase was strongly upregulated in all three triglyceride-treated groups, especially in the trilinolein group, further indicating a redox-related transcriptional response to the lipid-associated storage environment.

DNA metabolic process, DNA replication, and catalytic activity acting on DNA were enriched in the tricaprylin and trilinolein groups (Figure 5A,E). These terms are associated with DNA replication, repair, recombination, and processing [88,89]. In parallel, *parE*, encoding DNA topoisomerase IV subunit B, was upregulated in all three triglyceride-treated groups (Table 4), providing a gene-level example consistent with DNA-processing functions. Together, these findings suggest a transcriptional tendency toward maintenance of genetic-material stability after storage and rehydration. Glycosyltransferase activity was also enriched in all three groups, consistent with functions involved in extracellular polysaccharide, cell-wall polysaccharide, and other cell-surface glycosylated structures [90,91]. The concurrent upregulation of *dapA*, encoding 4-hydroxy-tetrahydrodipicolinate synthase, further indicated transcriptional regulation related to lysine, diaminopimelate, and peptidoglycan-precursor metabolism [92]. These two levels of evidence jointly point to cell-envelope-associated transcriptional regulation, although they do not represent the same biochemical pathway. Because extracellular polysaccharide content and cell-wall composition were not directly measured, these interpretations remain functional indications at the transcriptional level.

Overall, the representative upregulated genes provide gene-level examples consistent with the major enriched modules involving transport, redox responses, DNA-related processes, and cell-envelope-associated functions. These features were biologically consistent with the improved post-storage stress tolerance and may provide a physiological context for the membrane-integrity patterns observed after acid exposure, but they do not establish a direct causal relationship.

#### 3.6.3. GO Enrichment Analysis and Representative Downregulated Genes in Triglyceride-Treated Groups

GO enrichment analysis of downregulated genes showed that the three triglyceride-treated groups shared a broadly similar pattern involving basal metabolism, nitrogen-containing compound metabolism, cellular metabolism, and biosynthesis-related functions (Figure 5B,D,F). Compared with the oil-free control, genes associated with these growth-related metabolic and biosynthetic processes were enriched among the downregulated genes after storage, rehydration, and oil removal. Similar responses have been reported in lactic acid bacteria under environmental stresses, where rapid-growth and biosynthetic processes can be suppressed while resources are redirected toward stress tolerance and cellular maintenance [93]. In *L. plantarum*, olive-oil exposure has likewise been reported to induce a starvation-like transcriptional response, including repression of fatty-acid biosynthesis and related metabolic functions [94]. Although the lipid system and treatment conditions differed from those used here, these observations support interpretation of the present pattern as a relatively conservative, lipid-associated transcriptional state rather than a simple loss of cellular function.

Several downregulated terms with relatively high enrichment ratios were associated with ribosome structure, translation, tRNA/RNA-related catalytic activity, and nucleotide or cyclic-compound binding (Figure 5B,D,F). Consistently, *tuf*, *rnpA*, a tRNA synthetase class II domain-containing gene, and ribosomal protein S18 were all downregulated (Table 4), representing translation elongation, RNA processing, aminoacyl-tRNA synthesis, and ribosome structure, respectively [95,96]. Their coordinated decrease provided gene-level support for the enrichment of translation- and ribosome-related downregulated functions. Because protein synthesis is a major energy-consuming process, reduced transcription of these functions under environmental stress can contribute to lower energy expenditure and resource reallocation toward cellular maintenance [97,98]. This interpretation is consistent with the broader reduction in growth- and biosynthesis-associated functions described above, while remaining limited to the transcriptional level.

Meanwhile, *adhE* and class-II fructose–bisphosphate aldolase, involved in fermentation-related redox metabolism and glycolysis, were also downregulated (Table 4), providing gene-level examples consistent with the broader reduction in growth-associated metabolic activity [99]. Their direction of change complements the GO-level suppression of basal and cellular metabolic processes and links the selected representative genes directly to the enriched downregulated modules. Together, the GO enrichment and representative genes indicate coordinated downregulation of translation and central metabolic functions in the triglyceride-treated groups. These results should not be interpreted as direct measurements of protein-synthesis rate, ribosome activity, or metabolic flux.

## 4. Conclusions

Within the experimental conditions tested, triglyceride-associated protection of freeze-dried *L. plantarum* 124 showed different response patterns among the three triglyceride systems and across storage stages. A common protective window was identified at 25 °C for 30 d, within which all three triglycerides reduced viability loss. However, the stability of this protection diverged with fatty acid unsaturation. Tricaprylin, a saturated medium-chain triglyceride, provided the most stable protection, retaining high fatty acid chain integrity and generating no detectable oxidation products. In contrast, triolein and particularly trilinolein underwent progressive oxidation, which compromised protection during prolonged storage and became detrimental under accelerated conditions. Cells recovered from the protective-window samples exhibited improved acid, bile salts, and simulated digestive-fluid tolerance, accompanied by better-preserved membrane integrity. Transcriptomic analysis further revealed transcriptional patterns involving enhanced transport-, redox-, DNA-, and cell-envelope-related functions together with reduced translation- and growth-associated metabolism. These transcriptional patterns were associated with the improved post-storage stress-tolerance phenotype, but their causal role remains to be established.

Nevertheless, the protective window was narrow and condition-specific, highlighting a key limitation: the benefits of lipid matrices are easily erased by excessive oxidation. Because the statistical analyses were based on condition-specific treatment-versus-control comparisons, the observed differences among triglycerides and storage stages should be interpreted as response patterns rather than formally established structure or stage effects. Further work should focus on elucidating the molecular mechanisms by which lipid oxidation products interact with bacterial membranes and intracellular targets, preferably by combining metabolomics, lipidomics, and proteomics with genetic approaches. This study provides a basis for designing lipid-containing probiotic systems with controlled storage stability and stress tolerance.

## Figures and Tables

**Table 1:** Storage-induced log reductions, *p*-values, and differences in storage-induced log reduction at 25 °C. CK, oil-free control; LR, storage-induced log reduction; ΔLR, difference in storage-induced log reduction, calculated as the LR of the oil-free control minus that of the corresponding triglyceride-treated group. Positive ΔLR values indicate smaller viability losses than the oil-free control, whereas negative values indicate greater losses. Data are presented as mean ± standard deviation of three independent biological replicates (*n* = 3). LR values represent within-group changes relative to each group’s own 0 d viable count and should therefore be distinguished from the absolute viable counts presented in Figure 1.

Time (d)	CK LR	Tricaprylin			Triolein			Trilinolein		
		LR	ΔLR	*p*-Value vs. CK	LR	ΔLR	*p*-Value vs. CK	LR	ΔLR	*p*-Value vs. CK
15	0.19 ± 0.06	0.10 ± 0.02	0.10	0.02	0.14 ± 0.04	0.06	0.128	0.11 ± 0.04	0.09	0.032
30	0.60 ± 0.09	0.30 ± 0.10	0.29	0.003	0.24 ± 0.09	0.36	<0.001	0.24 ± 0.04	0.36	<0.001
45	0.80 ± 0.13	0.39 ± 0.09	0.42	0.002	0.61 ± 0.11	0.19	0.076	0.71 ± 0.11	0.10	0.309
60	1.29 ± 0.09	0.73 ± 0.12	0.56	-	1.14 ± 0.04	0.15	-	1.41 ± 0.15	−0.11	-
75	1.59 ± 0.11	1.12 ± 0.13	0.47	-	1.67 ± 0.04	−0.09	-	1.88 ± 0.17	−0.29	-
90	2.24 ± 0.07	2.02 ± 0.16	0.22	-	2.63 ± 0.10	−0.39	-	3.24 ± 0.20	−0.99	-

**Table 2:** Retention of parent fatty acid methyl esters in different triglyceride systems during storage.

Triglyceride System	Parent Fatty Acid Methyl Ester	Molecular Formula	Content at 0 h (μg/mg Oil)	Content at 25 °C for 30 d (μg/mg Oil)	Retention at 25 °C (%)	Content at 37 °C for 30 d (μg/mg Oil)	Retention at 37 °C (%)
Tricaprylin	Methyl octanoate	C_9_H_18_O_2_	942 ± 15	912 ± 14	96.8 ± 2.1	904 ± 4	96.0 ± 1.6
Triolein	Methyl oleate	C_19_H_36_O_2_	907 ± 13	859 ± 15	94.7 ± 2.2	778 ± 16	85.8 ± 2.1
Trilinolein	Methyl linoleate	C_19_H_34_O_2_	926 ± 10	771 ± 25	83.3 ± 2.9	563 ± 26	60.8 ± 2.9

**Table 3:** Representative fatty acid chain-derived oxidation and cleavage products tentatively identified after storage. ND, not detected.

Triglyceride System	Compound Name	Molecular Formula	Temperature	Match Score	Semi-Quantitative Value (μg/g Oil)
Triolein	Hexanal dimethyl acetal	C_8_H_18_O_2_	25 °C	77.6	0.32 ± 0.01
			37 °C	88.9	7.59 ± 0.36
	Nonanal dimethyl acetal	C_11_H_24_O_2_	25 °C	90	6.19 ± 0.35
			37 °C	89.4	20.37 ± 1.41
	Methyl nonanoate	C_10_H_20_O_2_	25 °C	81.2	1.20 ± 0.05
			37 °C	94.9	8.30 ± 0.60
	Dimethyl azelate	C_11_H_20_O_4_	25 °C	ND	ND
			37 °C	95.2	22.65 ± 1.91
Trilinolein	Hexanal dimethyl acetal	C_8_H_18_O_2_	25 °C	75.6	9.72 ± 0.96
			37 °C	90.5	27.71 ± 2.80
	Methyl octanoate	C_9_H_18_O_2_	25 °C	94.8	9.03 ± 0.39
			37 °C	96.4	61.32 ± 3.67
	Methyl 9-oxononanoate	C_10_H_18_O_3_	25 °C	ND	ND
			37 °C	79	3.75 ± 0.28
	Dimethyl azelate	C_11_H_20_O_4_	25 °C	89.9	2.86 ± 0.24
			37 °C	96.9	77.10 ± 6.95

**Table 4:** Representative differentially expressed genes related to GO-enriched functions in triglyceride-treated groups compared with the oil-free control. Genes were selected based on functional relevance and |log_2_FC| ≥ 1.5 in all three comparisons; data are presented as mean ± standard deviation (SD) of three independent biological replicates (*n* = 3), and the values represent log_2_FC of A, B, and C versus CK, respectively. CK represents the oil-free control, while A, B, and C represent tricaprylin, triolein, and trilinolein treatments, respectively.

Gene Name	Gene Description	Log_2_FC		
		A vs. CK	B vs. CK	C vs. CK
*srlE*	PTS glucitol/sorbitol transporter subunit IIB	1.50 ± 0.16	2.37 ± 0.51	2.10 ± 0.48
*dapA*	4-hydroxy-tetrahydrodipicolinate synthase	1.95 ± 0.19	1.80 ± 0.17	2.04 ± 0.17
*parE*	DNA topoisomerase IV subunit B	1.53 ± 0.21	2.01 ± 0.21	1.58 ± 0.21
*C7M33_RS04090*	SDR family oxidoreductase	4.57 ± 0.44	4.31 ± 1.20	4.94 ± 0.77
*C7M33_RS04925*	BCCT family transporter	2.78 ± 0.12	2.60 ± 0.07	2.65 ± 0.10
*C7M33_RS08405*	metal ABC transporter ATP-binding protein	2.23 ± 0.11	2.15 ± 0.12	2.13 ± 0.13
*tuf*	elongation factor	−1.53 ± 0.07	−1.76 ± 0.08	−1.71 ± 0.04
*rnpA*	ribonuclease P protein component	−1.93 ± 0.08	−2.01 ± 0.05	−2.10 ± 0.07
*adhE*	bifunctional acetaldehyde-CoA/alcohol dehydrogenase	−2.47 ± 0.20	−2.68 ± 0.35	−2.89 ± 0.39
*Novel00210*	tRNA synthetases class II core domain	−4.63 ± 0.23	−5.12 ± 0.51	−4.75 ± 0.41
*Novel00136*	ribosomal protein S18	−3.65 ± 0.67	−3.60 ± 0.73	−3.20 ± 0.48
*C7M33_RS07790*	class-II fructose-bisphosphate aldolase	−1.94 ± 0.06	−2.18 ± 0.10	−2.13 ± 0.07

## Data Availability

The original contributions presented in this study are included in the article/Supplementary Materials. Further inquiries can be directed to the corresponding author.

## Supplementary Materials

The following supporting information can be downloaded at https://www.mdpi.com/article/10.3390/foods15172952/s1, Figure S1. GC–MS total ion chromatograms of the three triglyceride systems during storage. (A) Tricaprylin system. (B) Triolein system. (C) Trilinolein system. Each panel includes chromatograms obtained at 0 h, after storage at 25 °C for 30 d, and after storage at 37 °C for 30 d. The chromatograms are provided as supporting analytical evidence, whereas the quantitative contents and retention values of the corresponding parent fatty acid methyl esters are reported in Table 2.
