# Supplementary material for: Structure-Dependent Triglyceride Protection of Freeze-Dried Lactiplantibacillus plantarum: Storage Protection Window, Lipid Oxidation, and Stress Adaptation
Source: Foods. 2026 Aug 22;15(17):2952. doi: 10.3390/foods15172952 (PMC13565162; doi:10.3390/foods15172952)
Supplement: Supplementary file 1 [file foods-15-02952-s001.zip › foods-4461081-supplementary.pdf]

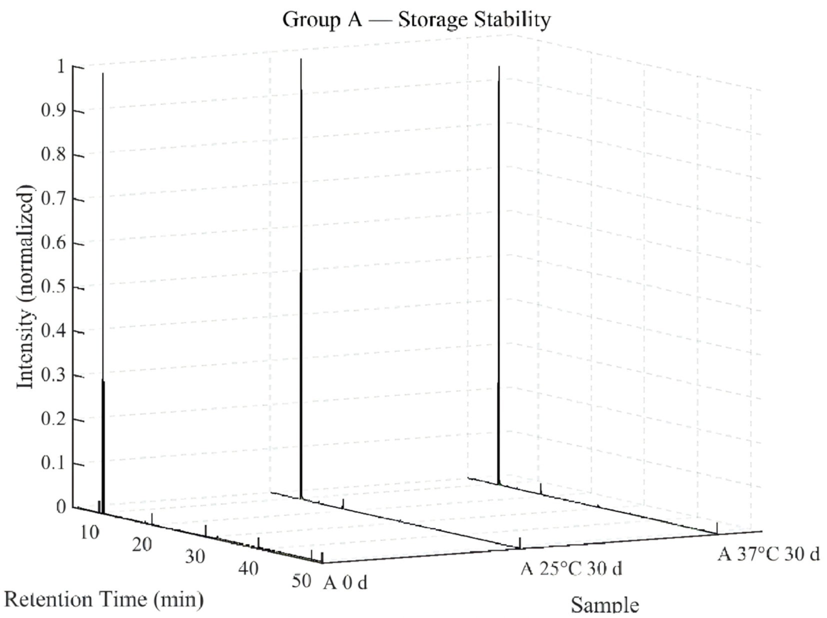
Group A — Storage Stability
Intensity (normalized)
0
0.1
0.2
0.3
0.4
0.5
0.6
0.7
0.8
0.9
1
10
20
30
40
50
Retention Time (min)
A 0 d
A 25°C 30 d
A 37°C 30 d
Sample

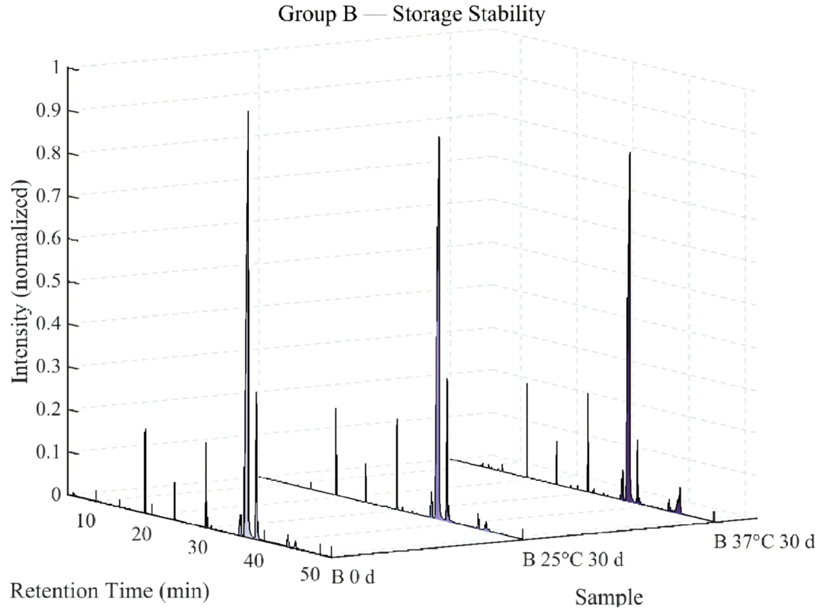
Group B — Storage Stability
Intensity (normalized)
0
0.1
0.2
0.3
0.4
0.5
0.6
0.7
0.8
0.9
1
10
20
30
40
50
Retention Time (min)
B 0 d
B 25°C 30 d
B 37°C 30 d
Sample

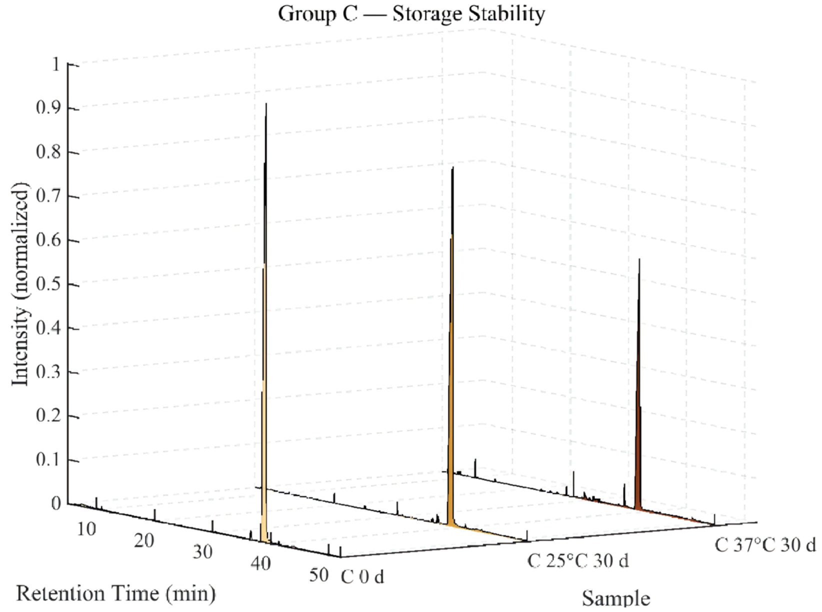
Group C — Storage Stability
Intensity (normalized)
0
0.1
0.2
0.3
0.4
0.5
0.6
0.7
0.8
0.9
1
10
20
30
40
50
Retention Time (min)
C 0 d
C 25°C 30 d
C 37°C 30 d
Sample

Figure S1. GC–MS total ion chromatograms of the three triglyceride systems during storage. (A) Tricaprylin system. (B) Triolein system. (C) Trilinolein system. Each panel includes chromatograms obtained at 0 h, after storage at 25°C for 30 d, and after storage at 37°C for 30 d. The chromatograms are provided as supporting analytical evidence, whereas the quantitative contents and retention values of the corresponding parent fatty acid methyl esters are reported in Table 2.
